# Survey of cryptic unstable transcripts in yeast

**DOI:** 10.1186/s12864-016-2622-5

**Published:** 2016-04-26

**Authors:** Jessica M. Vera, Robin D. Dowell

**Affiliations:** Department of Molecular, Cellular, and Developmental Biology, University of Colorado Boulder, Boulder, CO 80309 USA; BioFrontiers Institute, University of Colorado Boulder, Boulder, CO 80309 USA

**Keywords:** Cryptic unstable transcripts, CUTs, RNA-seq, Yeast, Rrp6, Transcriptome, Hidden Markov model, Nucleosome occupancy, *Saccharomyces cerevisiae*, *Saccharomyces paradoxus*

## Abstract

**Background:**

Cryptic unstable transcripts (CUTs) are a largely unexplored class of nuclear exosome degraded, non-coding RNAs in budding yeast*.* It is highly debated whether CUT transcription has a functional role in the cell or whether CUTs represent noise in the yeast transcriptome.

We sought to ascertain the extent of conserved CUT expression across a variety of *Saccharomyces* yeast strains to further understand and characterize the nature of CUT expression.

**Results:**

We sequenced the WT and *rrp6*Δ transcriptomes of three *S.cerevisiae* strains: S288c, Σ1278b, JAY291 and the *S.paradoxus* strain N17 and utilized a hidden Markov model to annotate CUTs in these four strains. Utilizing a four-way genomic alignment we identified a large population of CUTs with conserved syntenic expression across all four strains. By identifying configurations of gene-CUT pairs, where CUT expression originates from the gene 5’ or 3′ nucleosome free region, we observed distinct gene expression trends specific to these configurations which were most prevalent in the presence of conserved CUT expression. Divergent pairs correlate with higher expression of genes, and convergent pairs correlate with reduced gene expression.

**Conclusions:**

Our RNA-seq based method has greatly expanded upon previous CUT annotations in *S.cerevisiae* underscoring the extensive and pervasive nature of unstable transcription. Furthermore we provide the first assessment of conserved CUT expression in yeast and globally demonstrate possible modes of CUT-based regulation of gene expression.

**Electronic supplementary material:**

The online version of this article (doi:10.1186/s12864-016-2622-5) contains supplementary material, which is available to authorized users.

## Background

Numerous transcriptome studies have shown the eukaryotic genome to be highly expressed, revealing pervasive transcription of intergenic and unannotated, non-protein coding regions [1–4]. The discovery of unstable transcripts further adds to the complexity of the eukaryotic transcriptome. Cryptic unstable transcripts (CUTs) comprise a fraction of the unstable RNA population in yeast. These unstable, non-coding RNAs (ncRNAs) are RNA polymerase II transcribed and capped, but are terminated and polyadenylated by a non-canonical pathway involving the RNA binding proteins Nrd1, Nab3, and the poly(A) polymerase Trf4 of the TRAMP complex [5–8]. Following transcription termination, CUTs are rapidly degraded by the nuclear exosome [5] thereby rendering them virtually undetectable in wild type cells by traditional methodologies. Disrupting any step in this pathway will lead to CUT stabilization. However CUTs are customarily defined by dependency on Rrp6p nuclear exosome activity, and disrupting upstream steps, such as Nrd1p depletion or TRF4 deletion, result in extended or non-polyadenylated transcripts respectively [5, 8, 9], that do not accurately reflect CUTs as they would be in wildtype (WT) cells. Similar unstable ncRNAs have been identified in human cells by transient knock down of nuclear exosome components [10]. While many propose that CUTs are the result of spurious transcriptional activity and therefore rapidly degraded as a quality control mechanism [5, 6], others have argued for possible functional roles for CUTs or CUT expression in regulating gene expression [11, 12].

Historically regulation of gene expression has been attributed to sequence-specific DNA binding factors (transcription factors), transcription start site availability (via nucleosome positioning), and large co-activator complexes (such as Mediator). However it is increasingly clear that the act of transcription greatly influences the local chromatin environment through histone modifications and nucleosome repositioning [13–15]. Given the pervasive nature of CUT transcription and prevalent association with protein-coding genes, this transcriptional activity holds great potential to regulate gene expression. Although documented cases exist in which transcription of a CUT regulates the expression of a gene [12, 16–22], the functional basis of CUT expression remains highly debated and largely unexplored.

To date CUTs have only been identified in a single species of yeast, *Saccharomyces cerevisiae*, using the reference laboratory strain S288c [5, 11, 23]. We have utilized a hidden Markov model (HMM) to annotate CUTs from RNA-seq data in a variety of strains from *S.cerevisiae* and *S.paradoxus* thereby allowing us to identify conserved syntenic expression of CUTs between these two species which are predicted to have diverged 2–5 million years ago [24, 25]. It is well documented that important cellular functions are evolutionarily conserved, and we sought to identify the population of CUTs with conserved syntenic expression to gain insights into possible functional roles for CUT expression in yeast. Likewise, we can leverage CUT expression in other species of yeast to inform on the mechanisms underlying CUT expression.

## Results and discussion

### Explicit duration HMM identifies CUTs de novo from RNA-seq data

**Fig. 1:**
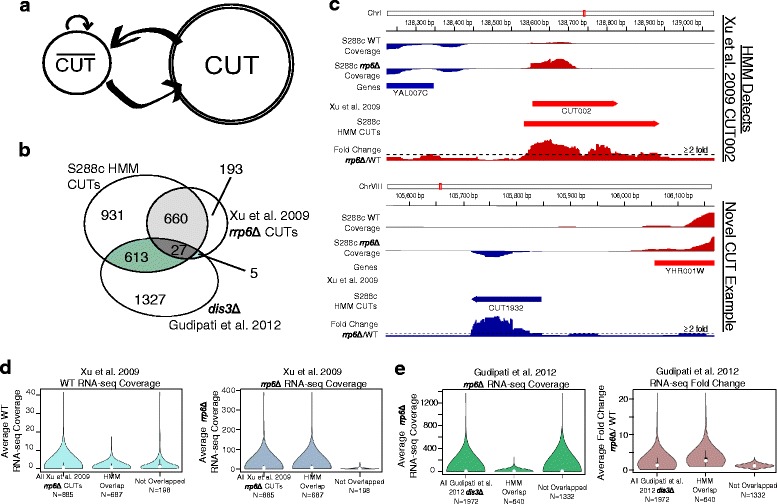
10-state HMM identifies CUTs de novo from RNA-seq. **a** Explicit duration HMM describing regions which have little to no fold change $\left(\overline{CUT}\right)$ and regions of elevated fold change in *rrp6*Δ relative to WT (CUT) as calculated from two biological replicates. **b** Venn diagram showing overlap between S288c CUTs annotations as determined by our HMM, *rrp6*Δ CUTs from Xu et al. [11], and *dis3*Δ transcripts from Gudipati et al. [31]. Minimum overlap of 25 % by length between annotations is required for positive matches. **c** IGV [53, 54] snapshots showing two examples of CUTs detected in S288c by our HMM. Top example shows previously identified Xu et al. CUT002 which is also identified by our HMM. Bottom example shows a novel CUT identified in this study. For each example, tracks are S288c WT RNA-seq coverage, S288c *rrp6*Δ RNA-seq coverage, annotated genes, Xu et al. [11] annotated CUTs, CUTs called by our HMM, and *rrp6*Δ/WT fold change within the region. Strand-specific data is color coded with Watson/plus strand in red and Crick/minus strand in blue. **d** Violin plots comparing the average S288c RNA-seq WT coverage and *rrp6*Δ/WT fold change from two biological replicates for all 885 possible Xu et al. [11] CUTs, the 687 CUTs overlapped by CUTs detected by our HMM, and the 198 remaining CUTs not overlapped by CUTs detected by our HMM. The Xu et al. [11] CUTs not identified in this study are presumably missed due to lack of stabilization in *rrp6*Δ. **e** Violin plots comparing the average S288c RNA-seq *rrp6*Δ and *rrp6*Δ/WT fold change from two biological replicates for all 1972 possible Gudipati et al. [31], the 640 transcripts overlapped by CUTs detected by our HMM, and the 1332 remaining transcripts not overlapped by CUTs detected by our HMM. The *dis3*Δ transcripts missed in previous *rrp6*Δ only, tiling array studies are presumably missed to limitations in tiling array sensitivity

To assess the extent of conserved CUT expression we utilized three strains of *S.cerevisiae*: S288c, Σ1278b, and JAY291, and a single strain of *S.paradoxus*: N17. In each strain background, biological duplicates of strand-specific RNA-seq libraries were prepared for wildtype (WT) and nuclear exosome mutant *rrp6*Δ backgrounds using the Illumina RNA ligation library protocol [26]. Reads were mapped to each strain’s respective genome assembly [27–29] (see Methods) and CUTs were identified by an explicit duration HMM (Fig. 1a) utilizing per nucleotide fold change values calculated from *rrp6*Δ and WT RNA-seq data (GEO accession GSE74028). Following previously established methods [11, 23] our HMM was parameterized to identify CUTs as regions of the transcriptome with elevated RNA-seq coverage in *rrp6*Δ approximately ≥ 2 fold over WT. Using the HMM we derived an initial set of raw CUT annotations that were subsequently filtered to remove specific nuclear exosome targeted transcripts such as snRNAs, snoRNAs, and rRNAs [5, 11], as well as expected hits resulting from genotypic differences in *rrp6*Δ strains relative to WT. Adjacent CUTs were merged based on an RT-PCR informed strategy (Additional file 1: Figure S1). Lastly we removed regions with low average *rrp6*Δ read coverage (see Methods), to reduce potential false positives, as well as any remaining regions less than 100 bp in length, in keeping with previously reported methods [5, 11].

In S288c a total of 2055 CUTs have been identified by our HMM. To benchmark and inform our HMM parameters we leveraged previous S288c *rrp6*Δ CUT annotations based on tiling arrays from Xu et al. [11]. In S288c we have identified 687 of 885 possible Xu et al. [11] CUTs (Fig. 1b), where a positive hit requires that our CUT annotation overlaps ≥ 25 % the length of the Xu et al. [11] annotation or vice versa (example in Fig. 1c), though overlap results were largely independent of the extent of overlap between features (Additional file 2: Figure S2A). In each case the number of positive hits is far greater than would be expected by chance (Additional file 2: Figure S2B). Those Xu et al. [11] CUTs missed by our HMM do not appear to be stabilized by disruption of nuclear exosome activity resulting from the loss of Rrp6p, though they do appear to be expressed in WT cells at levels equivalent to those CUTs we do identify and thus are not undetected due to low signal (Fig. 1d, Additional file 2: Figure S2C). Furthermore, of the Xu et al. [11] CUTs identified by our HMM, 523 overlap with the 622 Xu et al. [11] CUTs found upregulated in *rrp6*Δ by Fox et al. [30]. Additionally our HMM identified 1412 novel CUTs relative to previous Xu et al. [11] annotations (example Fig. 1c).

To further support our method of de novo CUT identification, we compared our CUTs to the *dis3*Δ transcripts from Gudipati et al. [31]. It was recently shown that the nuclear exosome subunit Dis3p/Rrp44p, which along with Rrp6p are the major catalytic components of the nuclear exosome, plays an active role in CUT degradation, showing a synergistic cooperation with Rrp6p [31]. While Gudipati et al. largely excluded the *rrp6*Δ Xu et al. [11] CUTs from their *dis3*Δ annotations, producing little overlap between those two data sets (Fig. 1b), we note that a large number, 640 of a possible 1972 *dis3*Δ transcripts (Fig. 1b), are detected by our HMM in an *rrp6*Δ background, far more than we would expect by chance (Additional file 2: Figure S2E). This demonstrates greater cooperation between the Dis3p and Rrp6p nuclear exosome subunits in the degradation of CUTs than was previously appreciated. Figure 1e and (Additional file 2: Figure S2F) shows that the *dis3*Δ transcripts identified in our study have an overall lower *rrp6*Δ read coverage than the *dis3*Δ transcripts as a whole, suggesting that these transcripts are lowly expressed and may have been missed previously due to the sensitivity limitations of hybridization-based assays [5, 11]. In contrast, the *dis3*Δ transcripts not identified by our study have an overall lower fold change in *rrp6*Δ relative to WT and are more likely to comprise a Dis3p-specific subset of nuclear exosome targets. These results underscore the need for high sensitivity methods for the detection of low abundance transcripts.

### CUTs appear to lack a defined 3′ nucleosome free region

**Fig. 2:**
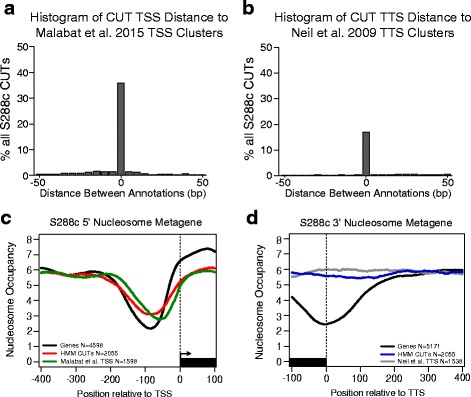
CUT start and stop sites concurrent with previous data and show distinct 3′ nucleosome structure. **a** Histogram showing the distribution of the distance between S288c CUT TSSs relative to Malabat et al. [32] CUT, intergenic, same sense, and antisense TSS clusters (see Methods). Histogram is only reporting distances for S288c CUTs that are within 50 bps of a TSS cluster. Bin widths are 5 bp. **b** Histogram showing the distribution of the distance between S288c CUT TSSs relative to Neil et al. [23] TTS clusters. Histogram is only reporting distances for S288c CUTs that are within 50 bps of a TTS cluster. Bin widths are 5 bp. **c** Metagene plot showing the average S288c nucleosome occupancy of a 500 bp window around the TSS for all genes with a 5′ UTR annotation (black), our HMM identified CUTs (red), and Malabat et al. [32] CUT TSS clusters (green). **d** Metagene plot showing the average S288c nucleosome occupancy of a 500 bp window around the TTS of all genes with a 3′ UTR annotation (black), our HMM identified CUTs (blue), and Neil et al. TTS clusters (grey)

To further asses the accuracy of our annotations, we compared our CUT 5′ and 3′ ends, as called by our HMM, to publically available transcription start site (TSS) and transcription termination site (TTS) annotations obtained by TSS sequencing and 3′ SAGE sequencing [23, 32] (Fig. 2a, b) performed in assorted *rrp6*Δ mutants. As many as 51 % of our S288c HMM CUT transcription start sites were found within 50 bp of Malabat et al. [32] TSS clusters. Likewise, 23 % of our S288c HMM CUT transcription termination sites were found within 50 bp of Neil et al. [23] TTS clusters. It has been previously established that CUTs, like other transcripts, have a 5′ nucleosome free region (NFR) upstream of the TSS [11]. Fig. 2c shows a metagene plot of 5′ nucleosome occupancy [33] in S288c comparing protein-coding genes with a 5′ UTR annotation [1], CUTs identified in this study, and CUT TSS clusters [32]. It is clear that CUTs identified by our HMM have a characteristic nucleosome depletion upstream of the TSS. However, when we compare the 3′ end of CUTs identified by our HMM to both protein-coding genes with a 3′ UTR annotation [1] and CUT TTS annotations [23], it is clear there is no distinct nucleosome depletion at the 3′ end (Fig. 2d) of CUTs. We observe a similar lack of 3′ nucleosome depletion for CUTs in Σ1278b and *S.paradoxus* (N17) (Additional file 3: Figure S3A), but were unable to make a similar observation for JAY291, as this strain lacks publically available nucleosome occupancy data. Conversely previously identified Xu et al. [11] CUTs showed a mild 3′ NFR, but we found this signal to be dominated by the set of CUTS that we failed to detect in our study (Additional file 3: Figure S3B). Along with snRNAs, snoRNAs, and to some degree rRNAs, CUT transcription termination and 3′ end processing is dependent on an alternative, non-canonical pathway that depends on the Nrd1-Nab3-Sen1 (NNS) complex [7]. Transcripts terminated through the NNS pathway have been described as terminating within a “zone” rather than a specific termination site, producing the varied and heterogeneous 3′ ends commonly observed for CUTs [34]. We acknowledge that CUT 3′ heterogeneity may affect the assessment of CUT 3′ NFRs by metagene analysis due to a lack of discrete and consistent TTS usage. We note that a similar difference between coding and non-coding gene 3′ nucleosome structure has also been observed in humans [35]. Interestingly, when we profile the 3′ nucleosome occupancy of yeast ncRNAs known as stable unannotated transcripts (SUTs) [11] (Additional file 4: Figure S4,) we see only moderate 3′ nucleosome depletion. While it is presumed that SUTs predominately utilize the same pathways as protein-coding genes for transcription termination and polyadenylation, it has also been shown that SUTs accumulate in NNS and nuclear exosome mutants [9, 11, 30, 36] demonstrating that these transcripts utilize the NNS pathway to some extent. The fact that SUTs show only a moderate well-defined 3′ NFR when compared to protein-coding genes may indicate greater utilization of the NNS pathway than was previously appreciated.

While it is clear that chromatin remodelers, DNA binding proteins, and A/T rich sequences are driving NFRs throughout the genome [33, 37–39], and that 5′ NFRs are regulating transcription initiation, the role of 3′ NFRs is less well understood. In humans, 3′ nucleosome depletion is hypothesized to regulate polyadenylation site selection and therefore subsequent 3′ end processing [40] of protein-coding genes. Transcription termination, 3′ end processing, and maturation of mRNAs is dependent on the cleavage and polyadenylation factor complex and comprises a pathway distinct from that of CUTs. Because they utilize distinct termination and 3′ end processing pathways it is possible that distinct 3′ nucleosome structures exist between mRNAs and CUTs. Our preliminary findings warrant further investigation regarding the role of 3′ nucleosomes in NNS-dependent transcription termination.

### A large set of CUTs show conserved expression between *S.cerevisiae* and *S.paradoxus*

**Fig. 3:**
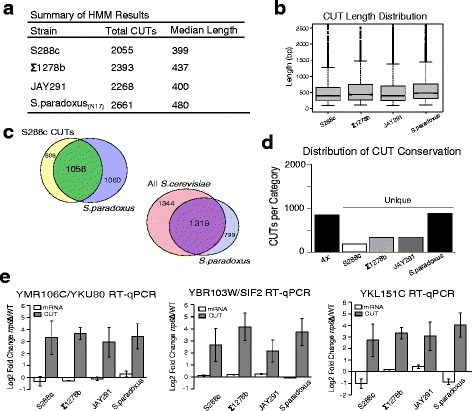
Assessment and validation of conserved CUT expression. **a** A summary of the HMM identified CUTs in each strain. **b** Box and whiskers plot showing CUT length distribution for each strain. We note that the y-axis range was limited to a maximum length of 2.5 kb for better comparison of the distributions across the strains. **c** Venn diagrams showing conserved CUT expression between the *S.cerevisiae* strain S288c and *S.paradoxus* (N17) and the conserved CUT expression between all *S.cerevisiae* strains (S288c, Σ1278b, and JAY291) and *S.paradoxus* (N17). **d** Distribution of CUTs with conserved syntenic expression across all four strains (4x) profiled or present in only one strain (unique). **e** RT-qPCR validation of three 4x conserved CUTs. In each case the candidate CUT is expressed antisense to an annotated gene and qPCR was performed strand-specifically with the same amplicon to distinguish between signal from the mRNA and the antisense CUT. Log2 fold change of *rrp6*Δ/WT was calculated after normalization to ACT1 (also acquired strand-specifically). In each case the CUT-specific strand shows a significant increase in transcript abundance in *rrp6*Δ relative to WT while the mRNA-specific strand shows little to no change, except with YKL151C mRNA. All qPCR was performed with biological triplicates and error bars denote standard deviation of fold change by coefficient of variation calculations

Having demonstrated that our HMM successfully annotates CUTs in S288c we then applied it to the remaining three strains: Σ1278b, JAY291, and N17 (Fig. 3a). Median CUT length in all four samples is approximately 400 nt, consistent with previous findings (Fig. 3a, b). As it remains largely unknown, we first sought to assess the extent of conserved CUT expression, here defined as detectable CUT expression within a syntenic genomic location. We used Pecan [41, 42] to perform a whole genome, multiple sequence alignment of the S288c, Σ1278b, JAY291, and N17 (*S.paradoxus*) genomes. The Pecan alignment generated a universal genomic coordinate system to which all CUT annotations were converted, allowing us to identify regions where detected CUTs overlapped across the strains. In order to be confident in identification of conserved expression, CUTs with no or poor 4-way alignment (see Methods) were excluded from subsequent analyses regarding CUT conservation, roughly excluding 20 % of all CUT annotations in each strain background. In total 64 % of S288c CUTs are conserved out to *S.paradoxus* (N17) (Fig. 3c). Alternatively we grouped all *S.cerevisiae* CUTs, 2663 in total, and found that about half are conserved out to *S.paradoxus* which corresponds to 62 % of all *S.paradoxus* CUTs (Fig. 3c). From our identified CUTs, 855 showed conserved syntenic expression across all four strains (labeled 4x in Fig. 3d) (Additional file 5: Table S1). Our set of 4x conserved CUTs include many well-known CUTs that are expressed at NRD1, IMD3, URA2, URA8, ADE12, and LEU4 [8, 12, 16]. We selected three 4x conserved CUTs, occurring at the SIF2/YBR103W, YKU80/YMR106C, and YKL151C loci, for validation by strand-specific quantitative PCR (RT-qPCR) (Fig. 3e). In each case strand-specificity was necessary for validation as the candidate CUTs are antisense to an expressed mRNA. To confirm the strand-specificity of our RT reactions, we measured signal from both strands of the amplicon, (i.e. both the CUT and the mRNA) which also allowed us to measure any changes in mRNA expression. In the case of both SIF2/YBR103W and YKU80/YMR106C the fold change from *rrp6*Δ to WT for the mRNA is relatively static (log2 fold ~ 0) while the CUT is elevated in *rrp6*Δ relative to WT. In the case of YKL151C, while again we see that the CUT is elevated in *rrp6*Δ, the YKL151C mRNA shows a moderate decrease in expression in both the S288c and N17 strains, though it remains unchanged in Σ1278b and JAY291.

In addition to 4x conserved CUTs, we identified CUT expression unique to each strain (Fig. 3d) and expression in intermediate patterns (in either 3 of 4 strains or 2 of 4 strains). We note that our designation of “strain unique” CUT expression is relative only to the four strains used in this study. As such the N17 (*S.paradoxus*) unique CUTs contain a combination of both strain and species unique CUTs whereas for the *S.cerevisiae* unique CUTs are predominantly strain specific, hence the greater number of unique CUTs for N17. We selected a small number of CUTs predicted in three of the four strains for validation by RT-qPCR in order to assess our false negative rate. Doing so, we failed to confirm the absence of the CUT in the fourth strain, implying that our method may have an appreciable false negative rate (Additional file 6: Figure S5). We note that many of these candidates pushed the lower bounds of qPCR detection, and we suspect that the fourth, unannotated CUT was likely missed by the HMM for similarly low abundance in our RNA-seq libraries. These results exemplify the difficulty in distinguishing between noise and true signal for low abundance RNAs even with the use of RNA-seq for their detection. Given these results, we suspect our assessment of conserved CUT expression to be conservative. However it is quite clear that a large, and potentially larger, subset of CUTs have conserved expression between these two species of yeast.

Using our 4-way genome alignment we sought to examine to what extent sequence conservation parallels conserved CUT expression patterns across the strains. (Additional file 7: Figure S6A) shows the distribution of average percent identity for 4x conserved CUTs compared to a random set of regions demonstrating that the sequence conservation of 4x conserved CUTs is no more or less than what can be expected by chance. CUT proximal promoters (300 or 50 bp upstream) have higher sequence conservation than corresponding regions of our randomized annotations. We note that the CUT and CUT promoter sequence conservation distributions are statistically distinct (*p*-value by two-sided KS test) possibly demonstrating distinct pressures for sequence conservation of these regions. Unique CUTs show a greater, but nonsignificant, variation in sequence conservation relative to 4x conserved CUTs, particularly in the promoter regions which may reflect sequence differences related to unique CUT expression (Additional file 7: Figure S6B). Admittedly, given that our four strains are closely related, the differences we see in sequence conservation are modest. Future studies at greater evolutionary distances are required to better elucidate the relationship between conserved CUT expression and sequence conservation.

### Distinct trends of gene expression correlate with CUT expression in specific architectures with genes

**Fig. 4:**
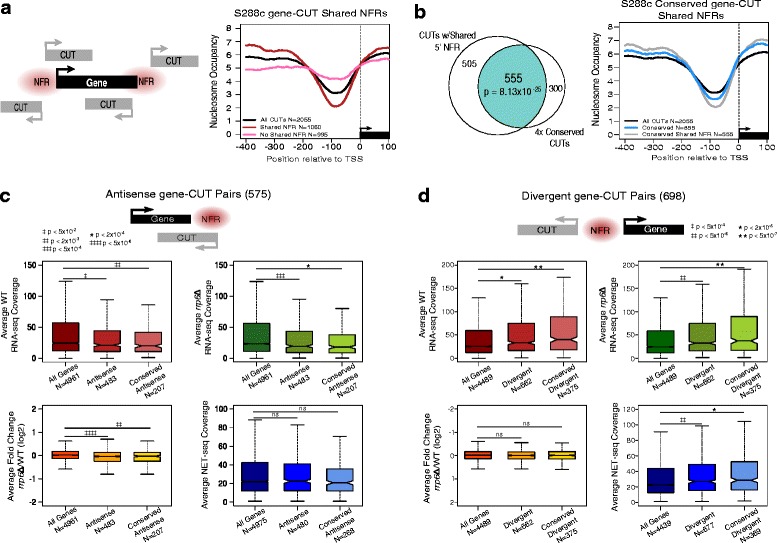
Distinct trends of gene expression correlate with CUT expression in specific architectures with genes. **a** *Left*: Schematic demonstrating the configurations in which a CUT can originate from a gene NFR. *Right*: Metagene plot of S288c nucleosome occupancy for a 500 bp window around the TSS of all S288c CUTs identified by our HMM (black), the subset of CUTs found to originate from a gene NFR (red), and the remaining CUTs that do not originate from a gene NFR (pink). **b** *Left*: Venn diagram of the overlap of CUTs that originate from a gene NFR and 4x conserved CUTs. *Right*: Metagene plot of S288c nucleosome occupancy for a 500 bp around the TSS of all S288c CUTs identified by our HMM (black), all of S288c 4x conserved CUTs (blue), and the 4x conserved CUTs that originate from a gene NFR (grey). **c** Examination of antisense gene-CUT pairs in S288c. Box and whisker plots showing the distribution of average WT (red), rrp6Δ (green), and log2 rrp6Δ/WT (orange) calculated from two S288c RNA-seq biological replicates, and NET-seq coverage from [4] (blue) for all expressed genes with a 3′ UTR annotation, those genes in antisense gene-CUT pairs, and the genes from the subset of antisense gene-CUT pairs with a 4x conserved CUT. All points outside the whiskers (outliers) are not displayed. All *p*-values are derived from the two-sided KS test. **d** Examination of divergent gene-CUT pairs in S288c. Box and whisker plots shows the distribution the average WT (red), rrp6Δ (green), and log2 rrp6Δ/WT (orange) calculated from two S288c RNA-seq biological replicates, and NET-seq coverage from [4] (blue) for all expressed genes with a 5′ UTR annotation, those genes in divergent gene-CUT pairs, and the genes from the subset of divergent gene-CUT pairs with a 4x conserved CUT. All points outside the whiskers (outliers) are not displayed. All *p*-values are derived from the two-sided KS test. Nonsignificant (ns) *p*-value ≥ 0.1

It has been suggested that spurious transcription at open chromatin leads to CUT expression [5], and indeed it has been shown that a large fraction of CUTs originate from the 5′ or 3′ NFR of protein-coding genes [11, 23]. In total 1060 (52 %) S288c CUTs identified by our HMM originate within either the 5′ or 3′ NFR of a gene (Fig. 4a). These CUTs show greater average depletion in 5′ nucleosome occupancy than CUTs that do not originate from a gene NFR (Fig. 4a). Interestingly the 4x conserved set of CUTs are over-enriched for CUTs that originate from a gene NFR (*p* = 8.13 ×10^−25^ by hypergeometric test) (Fig. 4b) and this enrichment is apparent as a moderate enrichment in 5′ nucleosome depletion of 4x conserved CUTs relative to all CUTs in S288c (Fig. 4b). We see a similar trend for increased 5′ nucleosome depletion for 4x conserved CUTs over all CUTs in both Σ1278b and *S.paradoxus* (N17) (Additional file 8: Figure S7).

We propose that CUTs that originate from or share a gene NFR are in a strong position to influence expression of the associated gene in *cis*. CUTs originating from the 3′ NFR of a gene could reduce gene expression via transcriptional inference [43] whereas CUTs originating from shared 5′ NFR regions may contribute to maintaining an open chromatin conformation [11] to aid gene expression. To test for possible CUT-based regulation of these genes genome-wide, we subdivided gene and CUT NFR sharing into two general configurations: convergent, overlapping gene-CUT pairs where the CUT 5′ NFR overlaps the gene 3′ NFR (subsequently referred to as antisense) and divergent, non-overlapping gene-CUT pairs that share a 5′ NFR (subsequently referred to simply as divergent) (Fig. 4c, d). We note that the remaining configurations, in which CUT transcription is same sense and overlapping with a gene, not only occur less frequently but are also are more difficult to analyze as we cannot distinguish read coverage between the two features (CUT and gene) and therefore cannot accurately assess transcript levels for either.

### Antisense CUT expression shows evidence of transcriptional interference on sense strand

First we examined antisense gene-CUT pairs, identifying 483 such pairs in S288c (Fig. 4c). We compared expression of the genes in these gene-CUT pairs to all expressed genes, excluding those with a same sense overlapping CUT over ≥ 50 % the length of the gene CDS. Overall the genes associated with antisense CUTs showed generally decreased expression compared to all expressed genes, a trend that is more pronounced when considering only the 4x conserved CUTs (Fig. 4c). This trend is lost, however, when we examine nascent transcription by NET-seq [4] (Fig. 4c, bottom right). This pattern is consistent with a model where CUTs impact the overlapping gene through transcriptional interference [43]. Because NET-seq detects nascent, actively transcribed RNA polymerase II RNAs, including nuclear exosome targeted transcripts before they are degraded, we are able to observe the impact of CUT expression on the nascent transcription of associated genes. By NET-seq it appears that antisense CUT expression does not repress active transcription of overlapping genes. Instead, reduced expression of genes in antisense gene-CUT pairs is limited to steady-state RNA levels (i.e. RNA-seq) suggesting that antisense CUT transcription is causing early termination of overlapping genes.

Several studies report anti-correlation between stable sense-antisense transcript expression [11, 23, 44] however we did not observe a correlation between CUT and gene RNA-seq or NET-seq expression levels, nor did we observe a correlation between CUT expression and gene repression levels, where repression was measured as the difference in gene NET-seq signal and WT RNA-seq signal. Similar to previous reports [36, 44] regarding stable sense-antisense pairs, we observed an overall greater reduction of gene expression in *rrp6*Δ compared to WT. While mechanisms of transcriptional interference do not require a stable interfering transcript [43], we speculate that stabilization of the interfering transcript upon loss of Rrp6p may result in increased gene repression through increased DNA:RNA hybrid formation [45, 46].

To determine if reduced gene expression in the presence of antisense CUT expression is conserved across strains we examined all antisense pairs containing 4x conserved CUTs in our remaining strains. We observed the same general trend of reduced expression for genes in these gene-CUT pairs, but this shift is not statistically significant by the two-sided KS test (Additional file 9: Figure S8). It is possible that this lack of statistical significance results from fewer total gene-CUT pairs in the remaining strains. In some cases we simply lack an annotation for the corresponding gene; in other cases the gene is not expressed and was thus removed from the analysis.

We have observed a trend for reduced expression of the genes found in antisense gene-CUT pairs similar to what is observed for stable sense-antisense pairs [44]. Antisense transcription is often found to elicit a negative effect on sense transcription via transcriptional interference, and has been widely studied in yeast [18, 36, 44, 47], but almost exclusively in the context of stable ncRNAs. Our results demonstrate that antisense CUTs elicit a negative effect on sense gene transcription in a manner consistent with stable ncRNAs and thus establish CUTs as possible sources of transcriptional interference.

### Divergent CUT expression correlates with higher gene expression

Next we examined divergent gene-CUT pairs, identifying 698 in S288c (Fig. 4d). We find that genes in this configuration have increased expression relative to all expressed genes and that this trend is more pronounced when looking only at those gene-CUT pairs with 4x conserved CUTs. We observed moderate gene ontology enrichment for various metabolic processes for genes found in divergent gene-CUT pairs, but this enrichment is lost when we only look at 4x conserved CUT pairs Additional file 10: (Table S2). Notably this trend of higher gene expression appears to originate at the level of transcription as it is observed in both nascent [4] and steady state RNA levels. This trend is consistent across all strains (Additional file 11: Figure S9). Additionally we did not observe a correlation between CUT expression and gene expression levels in S288c in any sequencing data set (data not shown). These results are consistent with a model where divergent expression of a CUT may help to maintain an open chromatin confirmation [11].

Next we wondered if increased gene expression is a general phenomenon of divergent transcripts or if this effect is specific to gene-CUT pairs. To address this we examined divergent gene-gene pairs, identifying 398 pairs, far fewer than gene-CUT divergent pairs despite a far greater number of protein coding genes overall suggesting a bias for CUTs in divergent transcript pairs with protein coding genes. When we compared the expression of divergent gene pairs to all expressed genes (Additional file 12: Figure S10) we did not find a significant difference in the expression distribution suggesting the effect seen in Fig. 4d is specific to CUTs.

Many have characterized bidirectional transcription, looking at both CUTs and stable ncRNAs [11, 23] but have failed to report on any observed effects on the expression of the associate genes. We hypothesized that divergent CUT expression from a shared NFR may help maintain the NFR thereby allowing for rapid and efficient expression of the associated gene and most likely benefitting higher expressed genes. Others have reported that long and deep NFRs commonly correlate to constitutive and highly expressed growth genes [24]. That genes found in divergent gene-CUT pairs are enriched for various metabolic processes is consistent with these previous findings. While we cannot rule out that CUT expression is an incidental result of higher expression at these genes, we note that we do not see divergent CUT expression at all highly expressed, or even the highest expressed genes. Additionally we see little correlation between CUT and gene expression levels further suggesting that CUT expression not a spurious result of leaky promoters of highly expressed genes. Strikingly divergent gene-gene pairs did not elicit the same expression trends observed in gene-CUT pairs in the same configuration. This further supports a role for divergent CUT expression in regulating the expression of associated genes and hints to the possibility of CUT-specific factors in mediating this trend.

## Conclusion

In this study, we used an explicit duration HMM to annotate CUTs from RNA-seq in an *rrp6*Δ background for a variety of yeast strains from the species *S.cerevisiae* and *S.paradoxus*. This allowed us make the first assessment of conserved intra- and interspecies CUT expression. Though our estimates appear conservative, we find that CUT expression is highly conserved within and between these two species of yeast despite the presence of sequence variation within upstream promoter regions. These findings warrant additional studies to assess CUT expression in other, more distantly related yeast species to better understand the relationships between DNA sequence and CUT expression. As many others have shown, CUT expression is commonly observed adjacent or overlapping with protein-coding genes [5, 8, 11, 23]. By identifying antisense and bidirectional gene-CUT pairs our work demonstrates that CUT expression is not only highly associated with protein-coding genes, but may also be regulating genes in a manner consistent with the location and orientation of CUT expression within gene-CUT pairs. Our work has additionally demonstrated CUTs and other NNS-terminated transcripts may have 3′ nucleosome structures distinct from that of protein-coding genes, warranting further investigation into the effect of termination mechanisms on nucleosome positioning.

## Methods

### Strain construction

Σ1278b WT and S288c (BY4741) WT were provided by the Fink lab. Σ1278b *rrp6*Δ and S288c *rrp6*Δ were provided by the Boone lab [28]. JAY291 WT was provided by Lucas Argueso [27]. We transformed JAY291 WT with the KanMX cassette from S288c *rrp6*Δ to delete RRP6 in JAY291. N17 WT was provided by the Fay lab, and transformed with a NatMX cassette to delete RRP6 in N17. See (Additional file 10: Table S3) for complete strain genotypes.

### Genome sequences and annotations

S288c genome and annotations are from the Saccharomyces Genome Database (SGD) S288c genome version 64 [29]. Σ1278b genome and annotations are available from Dowell et al. [28]. JAY291 genome and annotations are from the Duke 2009 [27] release, downloaded from SGD. We used a modified version of the JAY291 Duke 2009 assembly, where the reverse compliment of several contig sequences were used so as to match the orientation of homologous S288c sequences (Additional file 13). N17 genome and annotations were downloaded from the Sanger Welcome Trust FTP site as part of the Saccharomyces Genome Sequencing project [48].

### RNA-sequencing libraries

Cells were grown in YPD to an OD of 0.6. Total RNA was isolated via hot acid phenol method and DNAse treated with Promega DNAse RQ1 to remove contaminating DNA. Poly(A) RNA was isolated using either a single round of Qiagen oligotex mRNA isolation kit or two rounds of Dyna bead mRNA isolation kit. Strand specific RNA-seq libraries were constructed from 500 ng of poly(A) RNA using the Illumina RNA ligation library protocol from [26]. We sequenced, by Illumina HiSeq, biological duplicates of each sample. To remove any contaminating rRNA reads, we first used Bowtie v0.12.7 [49] to map reads to a single repeat of the rDNA locus allowing two mismatches. The remaining reads were mapped uniquely to the genome sequence of each respective strain allowing up to two mismatches. See (Additional file 10: Table S4) for a summary of read mapping results. Per nucleotide read coverage was obtained using BEDTools [50], corrected for read first nucleotide biases and read mappability, and then normalized by the tens of millions of mapped reads per sample. Per nucleotide coverage was averaged across replicates. Fold change from *rrp6*Δ to WT was calculated for every nucleotide in the genome using bias corrected coverage values. A Laplace prior (+1) was added to all coverage values to avoid division by zero when calculating the per nucleotide fold change.

### Explicit duration hidden Markov model

We developed an explicit duration hidden Markov model (HMM) to analyze per nucleotide *rrp6*Δ/WT RNA-seq fold change signal (Fig. 1a) using the Matlab HMM toolkit (MATLAB 2012b, The MathWorks Inc., Natick, MA, 2012). The HMM consists of two main states, one parameterized to non-elevated regions of the transcriptome (i.e. not CUTs) and one for elevated (approximately ≥ 2 fold) regions of the transcriptome (i.e. CUTs). Specifically we expanded the CUT state into nine identical sub-states with unidirectional movement through the model (Additional file 14: Figure S11) thereby setting the minimum length of a CUT to nine nucleotides and producing a 10-State model that approximates a hidden semi-Markov model [51]. This allowed us to deviate from the exponential duration modelling of traditional HMMs and produce CUT annotations with a length distribution that better approximated previous studies [5, 11]. We note that when the model is used to generate representative sequences, the CUT state of the model produced sequences that are generally long (>34,000 bp) reflecting our bias to identify long regions of relatively consistent elevated coverage. Per nucleotide fold change values were converted to discrete values for analysis by our HMM as necessitated by the Matlab toolkit (Additional file 10: Table S5). Transition and emission probabilities are available in (Additional file 10: Tables S4, S5).

### CUT identification

From the HMM we derived an initial set of raw CUT annotations. These raw annotations were filtered to remove snRNAs, snoRNAs, and rRNAs as well as expected hits resulting from genotypic differences in *rrp6*Δ strains relative to WT. Any remaining regions within 450 bp were merged together into a single annotation. Regions with average *rrp6*Δ read coverage less than the upper two-thirds of all nonzero coverage values for that strain and any regions less than 100 nt in length also were removed. Final CUT annotations are available from the GEO repository under accession number GSE74028 at http://www.ncbi.nlm.nih.gov/geo

### Annotation overlap and significance test

We used IntersectBed [50] to quantify the extent of overlap between our HMM S288c CUT annotations and other data sets (Fig. 1b) requiring overlap of ≥25 % the length of either annotation. Because we removed raw HMM CUT annotations that overlapped snRNAs, snoRNAs, and rRNAs, we likewise removed any annotations from Xu et al. [11] and Gudipati et al. [31] that overlapped the removed raw HMM CUTs in S288c to properly reflect the extent of overlap between these data sets and our S288c CUTs. Hence only 885 of a total 925 Xu et al. [11] CUTs and 1972 of a total 2032 Gudipati et al. [31] *dis3*Δ transcripts were used in subsequent overlap analyses. To determine statistical significant we randomly sampled genomic regions with the same length distribution as S288c identified CUTs. After 200 iterations, overlap of these randomly sampled regions and previously annotated CUTs or *dis3*Δ transcripts approximate a normal distribution (Additional file 2: Figure S2B,E). We use two standard deviations from the mean to assess significance within our CUT annotations.

### Nucleosome occupancy and metagene analysis

For S288c nucleosome occupancy we used summarized nucleosome occupancy from Field et al. [33] data available from the SGD website. For Σ1278b and N17 we mapped the raw reads from Tsankov et al. [24] according to their methods with the exception that we used the N17 *S.paradoxus* genome instead of NRRLY-17217 used in their study. Metagene plots were constructed by averaging the nucleosome occupancy for each base pair in a 500 bp window for all annotations in the analyzed data sets.

### CUT transcription start site comparisons

The Malabat et al. [32] study identified TSS clusters in various mutant backgrounds including *rrp6*Δ. TSS clusters were sorted and grouped according to their relative positions to annotated features. Since clusters assigned to CUTs required overlap with previous CUT annotations, we included all antisense, same sense, and intergenic (i.e. A, B, and I) clusters with an *rrp6*Δ/WT fold change ≥ 1.5 as calculated in their study.

### Pecan whole genome alignment

We used Pecan version 0.9 [41, 42] to generate a four-way whole genome multiple sequence alignment of the S288c, Σ1278b, JAY291, and N17 genomes. As the JAY291 genome is currently only available in a contig assembly [27], we first used BLAT to find the single best hit for each contig to the S288c genome in order to produce a pseudo-genome assembly as required by Pecan (Additional file 13).

### Conserved CUT expression

First we converted all CUT annotations from strain-specific coordinates to the 4-way alignment coordinate system. Then we calculated a histogram of CUT annotations along the 4-way alignment and all continuous regions ≥ 1 in the histogram were selected. The total histogram signal over these selected regions was averaged and used to determine the total number of CUTs overlapping that region. Regions with an average histogram signal > 4 denoted 4x conserved CUT expression. We identified 208 regions where the CUT annotations were incongruent across the four strains and applied hand edits to resolve these incongruences where possible. Additionally, we examined those CUTs in 3 of the 4 strains and if the CUT is missed in the fourth strain by our filtering procedure (i.e. the fourth strain has a CUT in the raw HMM output) we brought back the filtered CUT annotation and considered these to be 4X conserved CUTs. The resulting changes in CUT annotations are reflected in summaries reported in Fig. 3a. After removing those CUTs with indels (relative to the four-way alignment) for more than 25 % the length of the CUT, we derived the conserved expression results reported in Fig. 3c, d. In the case of unique CUTs (Fig. 3d) we only reported those CUTs that did not overlap a raw (but removed) annotation in either of other strains. To determine the significance of our CUT conservation analysis we randomized CUT annotations in all four strains to assess the chance of CUT conservation simply by chance. With 200 iterations, little to no random 4x conserved CUTs were found (Additional file 15: Figure S12).

### CUT expression validation by RT-qPCR

We selected candidate CUTs that were novel to our study relative to Xu et al. [11] however in some cases candidates were also identified by Gudipati et al. [31] as *dis3*Δ transcripts. To validate CUTs identified by the HMM we performed strand specific RT-qPCR using a 5′ tagged gene-specific RT primer [52] for cDNA synthesis of DNAsed, total RNA. In many cases strand specificity was necessary to distinguish CUT transcripts in the presence of overlapping, antisense mRNAs. Tagged RT primer distinguishes primer-specific cDNA from false primed cDNA that frequently occurs between overlapping, antisense transcripts. Subsequent PCR reactions used a universal forward primer complimentary to the RT tag and a gene specific reverse primer. Primer sequences can be found in Additional file 16. In some cases it was necessary to use the tagged RT primer as the forward primer during qPCR to avoid primer dimers between the universal forward primer and the gene-specific reverse primer. ACT1 was used as a normalizing endogenous control and was also measured strand specifically. A few candidates did not require strand-specific RT-qPCR (see Additional file 16). These samples instead used random hexamer RT primers and gene-specific qPCR primers. Fermentas Maxima Reverse Transcriptase was used for all RT reactions. Three biological replicates were grown to O.D. 0.6 in YDP and total RNA was isolated by hot acid phenol method and DNAse treated with Promega DNAse RQ1.

### NFR sharing between CUTs and protein-coding genes

Metagene plots in Fig. 2c, d show the general location of the 5′ NFR ranging from -200 to 0 bp from the transcription start site and the 3′ NFR ranging from +100 to -100 from the transcription termination site. We annotated these regions for each gene where corresponding untranslated region annotations were available [1]. We annotated CUT 5′ NFRs in the same fashion. We considered potential instances of NFR sharing when the CUT 5′ NFR annotation overlapped ≥50 % (minimum 100 bp) the length of a gene 5′ or 3′ NFR.

### Ethics

Not applicable.

### Consent to publish

Not applicable.

### Data availability

The raw and processed sequencing data along with all final HMM derived CUT annotations from this article are available in the GEO omnibus repository under accession number GSE74028 at http://www.ncbi.nlm.nih.gov/geo

## Additional files

Additional file 1: Figure S1. RT-PCR validation of raw CUT annotations merging strategy. Three candidate regions selected to determine whether adjacent CUT regions, supported by calls in multiple strains, should be merged in post processing. Candidates tested are located at the A) YNL299W/TRF5 locus B) YBR117C/TKL2 locus and C) YNL117W/MLS1 locus. In each case strand-specific RT primers were used to generate cDNA and PCR was performed to produce an amplicon that spans the gap in the raw annotations. Left: An IGV [53, 54] snapshot with tracks showing the gene, our raw CUT, and our final CUT annotations for the strains S288c, Σ1278b, and JAY291 after conversion to the 4-way Pecan alignment (see Methods). Additionally we show the location of each primer used and the resultant amplicon of a positive merge result. Strand-specific data is color coded with Watson/plus strand in red and Crick/minus strand in blue. Right: 2 % agarose gel showing RT-PCR results. For each candidate we designed two primer pairs with each pair located on either side of the gap between raw CUT annotations as identified by our HMM. We generated strand-specific cDNA from both WT and *rrp6*Δ total RNA samples with each reverse primer and performed PCR on these cDNA with F1/R2 primer pair. F1/R2 primers should produce a merge amplicon product only if the candidate CUT is a single transcript spanning the gap in raw CUT annotations. Amplification in R1 primed cDNA served as a negative control, as amplification should only occur in R1 primed cDNA; this also helped to confirm strand-specificity. We included genomic positive control, a no primer control (NPC) RT sample to distinguish false-primed cDNAs (denoted with *), and a no template control (NTC) to distinguish primer dimers. (PDF 8940 kb) Additional file 2: Figure S2. S288c HMM CUT comparison to Xu et al. [11] and Gudipati et al. [31] annotations. Comparisons of S288c CUTs identified by our HMM and Xu et al. [11] CUTs or Gudipati et al. [31] *dis3*Δ transcripts. Extent to which minimum overlap influences number of features concordant between HMM detected CUTs and A) Xu et al. [11] CUTs. B) Overlap is more than would be expected by chance. S288c CUT annotations were randomized (see Methods) and the number of features overlapped in each data set was collected over 200 iterations and plotted as a histogram. The average number of features overlapped after 200 iterations, with error bars denoting standard deviation, is plotted for comparison to actual S288c overlap results. Actual S288c CUTs overlap is greater than 2 standard deviations from random trials. C) Violin plots as seen in Fig. 1 d showing average RNA-seq fold change for all Xu et al. [11] CUTs, Xu et al. [11] CUTs overlapped by CUT identified by our HMM, and Xu et al. [11] CUTs missed by our study where we observe equivalent expression in WT and *rrp6*Δ backgrounds. (D-F) Similar comparison for Gudipati et al. [31] *dis3*Δ transcripts. (PDF 54 kb) Additional file 3: Figure S3. CUTs appear to lack a 3′ NFR. A) Metagene plot showing the average nucleosome occupancy of a 500 bp window around the TTS of all S288c(blue), Σ1278b(yellow), and *S.paradoxus*_N17_(teal) CUTs identified by our HMM. For comparison across strains, nucleosome occupancy was normalized by the average nucleosome occupancy per base pair in each strain. Like S288c CUTs, we see do not see 3′ nucleosome depletion in our other strains for which nucleosome occupancy data is available. B) Left: Metagene plot showing the average S288c nucleosome occupancy of a 500 bp window around the TTS of all genes with a 3′ UTR annotation (black), our HMM identified CUTs (blue), Neil et al. 2009 TTS clusters (grey), and Xu et al. [11] CUTs (pink). Moderate 3′ nucleosome depletion can be seen for Xu et al. CUTs 2009. Right: When we split the Xu et al. [11] CUT annotations into two groups, those overlapped by an S288c CUT identified by our HMM (maroon), and those that are not (lilac), we see distinct nucleosome occupancy patterns for the two groups. Those Xu et al. [11] CUTs that overlap an S288c CUT identified by our HMM also appear to lack a 3′NFR and the moderate depletion previously seen in the left graph is largely restricted to those Xu et al. [11] CUTS that we failed to detect and which also appear to be stable, albeit lowly expressed RNAs (see Fig. 1d and Additional file 2: Figure S2C). (PDF 70 kb) Additional file 4: Figure S4. ncRNAs have moderate 3′ nucleosome depletion. Metagene plot showing the average S288c nucleosome occupancy of a 500 bp window around the TTS of all genes with a 3′ UTR annotation (black), our HMM identified CUTs (blue), Neil et al. 2009 TTS clusters (grey), and ncRNAs (green) also known as stable unannotated transcripts (SUTs) from Xu et al. [11]. ncRNAs show moderate 3′ nucleosome depletion within the same 200 bp region where genes have a strong 3′ NFR producing a nucleosome occupancy pattern that is distinct from both CUTs and genes. (PDF 33 kb) Additional file 5: Table S1. 4x Conserved CUT Annotations. A table containing the strain-specific genomic coordinates for all 855 4x conserved CUTs identified in this study. (XLSX 134 kb) Additional file 6: Figure S5. Assessment of HMM false negative rate by RT-qPCR. RT-qPCR of CUTs expressed in three out of four strains (3x CUTs). For simplification candidates are named based on closest or overlapping protein-coding gene annotations (x-axis). Candidates are grouped and labeled (above the bar plot) according to the strain that lacks the corresponding CUT annotation. RT-qPCR was performed either strand-specifically or non-strand specifically depending on the presence of overlapping antisense gene annotations (see Methods; Additional file 16). Log2 fold change of *rrp6*Δ/WT was calculated after normalization to ACT1. The red dashed line marks two-fold cutoff. In all but one instance, JAY291 YNL146C-A, the “missing” CUT shows elevated expression, as seen in the remaining strains. All qPCR was performed with biological triplicates and error bars denote standard deviation of fold change by coefficient of variation calculations. (PDF 33 kb) Additional file 7: Figure S6. Sequence conservation of CUTs. A) Violin plots showing the average sequence conservation, calculated from our 4-way genome alignment, of all 4x conserved CUTs, 300 bp upstream and 50 bp upstream promoters (red), and compared to the average percent identity of a randomized set of annotations (grey) that recapitulates the 4x conserved CUTs in length and frequency. We used the S288c start coordinate and the longest stop coordinate as the start and stop coordinates for the 4x conserved CUTs when calculating average percent identity. Included are all *p*-values < 0.1 obtained by the two-sided KS test. B) Violin plots showing the average sequence conservation, calculated from our 4-way genome alignment, of the CUTs unique to each strain and the 300 bp upstream and 50 bp upstream promoters. Included are all *p*-values < 0.1 obtained by the two-sided KS test. (PDF 56 kb) Additional file 8: Figure S7. 4x conserved CUTs show increased 5′ nucleosome depletion relative to all CUTs. Metagene plot showing the average nucleosome occupancy in A)S288c, B)Σ1278b, and C)N17 of a 500 bp window around the TSS for all CUTs identified by our HMM in the respective strain (black) and all 4x conserved CUTs as annotated in each respective strain (grey). (PDF 54 kb) Additional file 9: Figure S8. Conserved antisense gene-CUT pairs in Σ1278b, JAY291, and *S.paradoxus.* Examination of antisense gene-CUT pairs containing a 4x conserved CUT. Box and whisker plots shows the distribution the average WT RNA-seq coverage (red), *rrp6*Δ RNA-seq coverage (green), log2 *rrp6*Δ/WT RNA-seq fold change (orange) for all expressed genes with a 3′ UTR annotation and the subset of genes from antisense gene-CUT pairs with a 4x conserved CUT in A) Σ1278b, B) JAY291, and C) *S.paradoxus*. All points outside the whiskers (outliers) are not displayed. All *p*-values are derived from the two-sided KS test. Nonsignificant (ns) *p*-value ≥ 0.1. (PDF 46 kb) Additional file 10: Table S2. Divergent gene-CUT pairs enriched for metabolic process genes. A total of 698 divergent gene-CUT pairs were identified in S288c. The subset of genes in these gene-CUT pairs are enriched for various metabolic processing gene ontologies (GO). *P*-values are based on the hypergeometric test after Holm-Bonferroni correction using the default background from YeastMine http://yeastmine.yeastgenome.org. The total number of genes in each GO category is listed far right. **Table S3**. Strains used in this study. A table describing the genotype and mating type of strains used in this study. **Table S4**. Summary of RNA-seq Read Mapping Results. A table summarizing the read mapping results for each RNA-seq library used in this study. Reported values for total mapped reads corresponds to all uniquely mapped reads after rRNA read removal. **Table S5**. Fold change conversion to discrete values. The Matlab HMM Toolkit only accepts discrete emission values. Per nucleotide *rrp6*Δ/WT fold change values were converted to a discrete value according to the table above. **Table S6**. HMM emission probabilities. The HMM emission probability for each discrete *rrp6*Δ/WT RNA-seq fold change value (see Table S3) for states 1–10. Because states 2–10 have the same emission probabilities we only show a single iteration of these emission probabilities for simplification. **Table S7**. HMM transition probabilities. The HMM transition probabilities for states 1–10. Movement through the HM is unidirectional and only two transition probabilities exist for each state. (XLSX 19 kb) Additional file 11: Figure S9. Conserved divergent gene-CUT pairs in Σ1278b, JAY291, and S.paradoxus. Examination of divergent gene-CUT pairs containing a 4x conserved CUT. Box and whisker plots shows the distribution the average WT RNA-seq coverage (red), *rrp6*Δ RNA-seq coverage (green), log2 *rrp6*Δ/WT RNA-seq fold change (orange) for all expressed genes with a 5′ UTR annotation and the subset of genes from antisense gene-CUT pairs with a 4x conserved CUT in A) Σ1278b, B) JAY291, and C) *S.paradoxus* (N17). All points outside the whiskers (outliers) are not displayed. All *p*-values are derived from the two-sided KS test. (PDF 46 kb) Additional file 12: Figure S10. Divergent gene-gene pairs in S288c. Examination of divergent gene-gene pairs in S288c. Box and whisker plots shows the distribution the average WT RNA-seq coverage (red), *rrp6*Δ RNA-seq coverage (green), log2 *rrp6*Δ/WT RNA-seq fold change (orange) for all expressed genes with a 5′ UTR annotation and the subset of genes from gene-gene pairs. Gene set 1 and gene set 2 are derived from the two separate genes from each gene-gene pair where gene 1 is also on the crick strand as shown in the schematic. All points outside the whiskers (outliers) are not displayed. All *p*-values are derived from the two-sided KS test. Nonsignificant (ns) *p*-value ≥ 0.1. (PDF 32 kb) Additional file 13: JAY291 Pseudo-genome Assembly. We used BLAT to find the single best hit for each JAY291 contig to the S288c genome in order to produce a pseudo-genome assembly as required by Pecan. This table lists the JAY291 contigs associated with each S288c chromosome in syntenic order. Asterisks denote contigs for which the reverse compliment sequence was used (see Methods). (XLSX 16 kb) Additional file 14: Figure S11. 10-state explicit duration HMM. A state diagram of our explicit duration HMM showing expansion of state 2 into nine equivalent sub-states. The first state is parameterized to non-elevated regions of the transcriptome (i.e. not CUTs) and the remaining states are parameterized for elevated (approximately ≥ 2 fold) regions of the transcriptome (i.e. CUTs). We expanded the CUT state into nine identical sub-states with unidirectional movement through the model thereby setting the minimum length of a CUT to nine nucleotides and producing a 10-State model that approximates a hidden semi-Markov model [51]. (PDF 37 kb) Additional file 15: Figure S12. Results of Randomized CUT Conservation Analysis. To determine the significance of our CUT conservation analysis we randomized CUT annotations in all four strains to assess the chance of CUT conservation simply by chance. A) Venn diagrams as seen in Fig. 3C showing the average and standard deviation of conserved CUT expression between the S.cerevisiae strain S288c and S.paradoxus (N17) and the conserved CUT expression between all S.cerevisiae strains (S288c, Σ1278b, and JAY291) and S.paradoxus (N17) after 200 randomized trials. B) Bar graph showing the actual total number of 4x conserved CUTs as found by our study and the average and standard deviation of the total number of 4x conserved CUTs after 200 randomized trials. (PDF 42 kb) Additional file 16: Primers Used for RT-qPCR. RT-qPCR primer names and sequences. Primers are specific to either *S.cerevisiae* (S.cere) or *S.paradoxus* (S.para), but in some cases could be used in either species background. All primers were named for the nearest or overlapping gene annotation. Those primers labeled “–T” denote the presence of the unique 5′ tagged used for strand-specificity in RT reactions. An asterisk in the final column denotes candidates not requiring strand-specific RT-qPCR; for these candidates qPCR was performed on random hexamer primed cDNA (see Methods). (XLSX 15 kb)

## Abbreviations

ncRNA: non-coding RNA
NFR: nucleosome free region
NNS: Nrd1-Nab3-Sen1 complex
RNA-seq: RNA sequencing
RT: reverse transcription/transcriptase
RT-qPCR: reverse transcribed quantitative polymerase chain reaction
TSS: transcription start site
TTS: transcription termination site
