# Supplementary figures and images for: Survey of cryptic unstable transcripts in yeast
Source: BMC Genomics. 2016 Apr 26;17:305. doi: 10.1186/s12864-016-2622-5 (PMC4845318; doi:10.1186/s12864-016-2622-5)

**A.**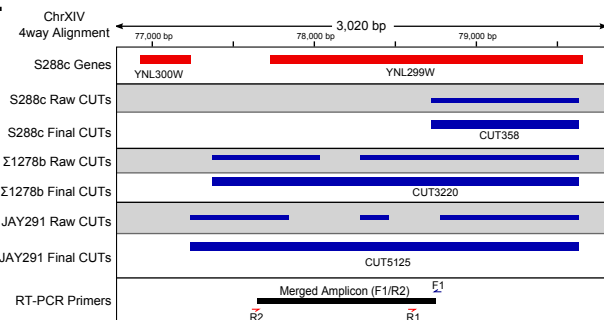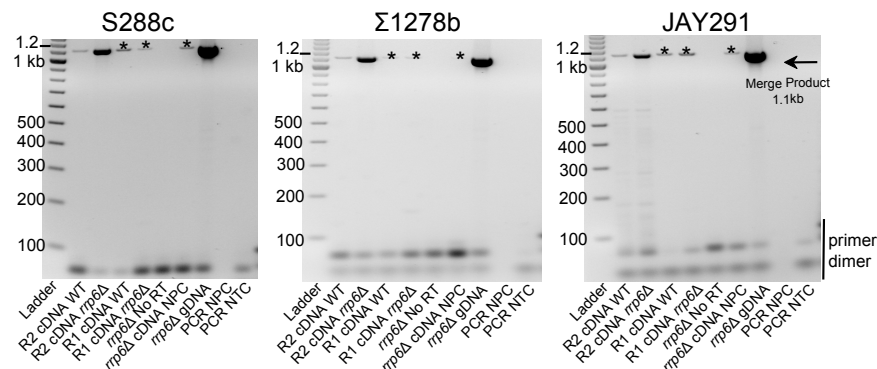**B.**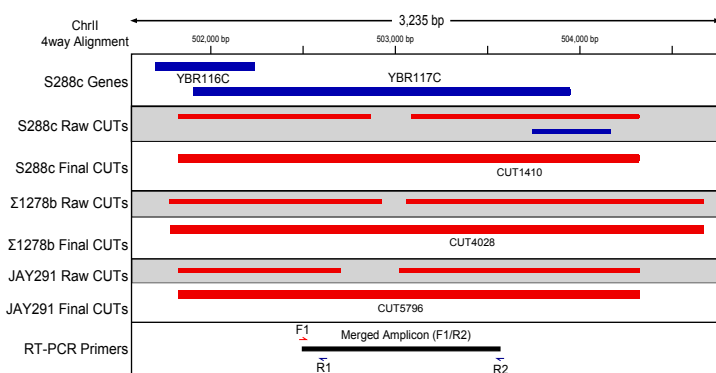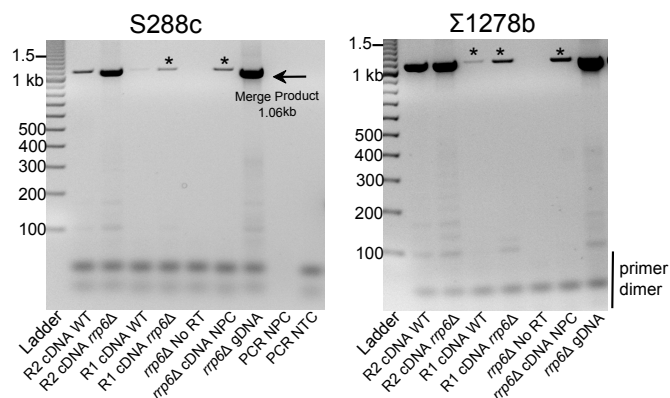**C.**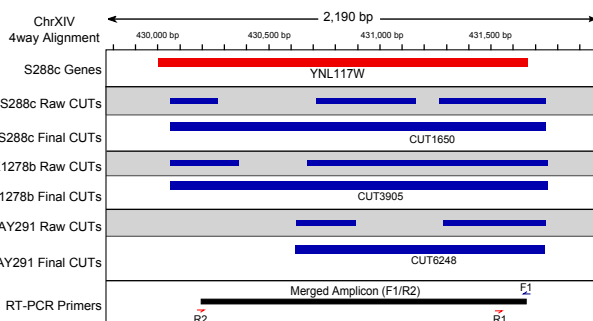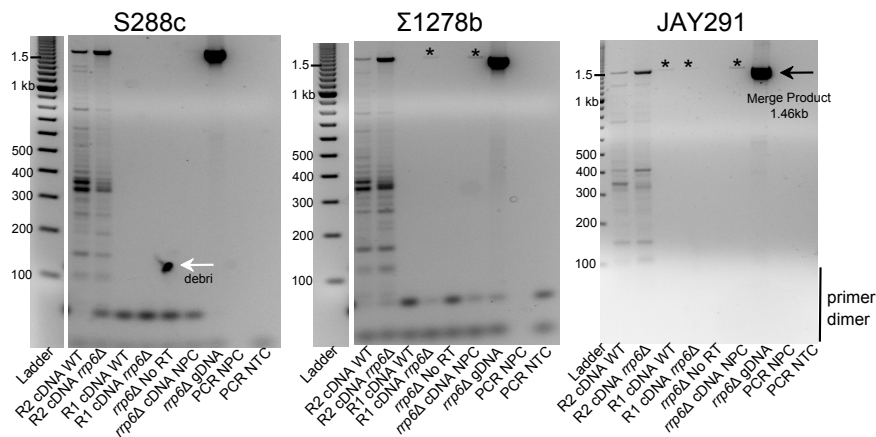

Supplement: Additional file 1: Figure S1. — RT-PCR validation of raw CUT annotations merging strategy. Three candidate regions selected to determine whether adjacent CUT regions, supported by calls in multiple strains, should be merged in post processing. Candidates tested are located at the A) YNL299W/TRF5 locus B) YBR117C/TKL2 locus and C) YNL117W/MLS1 locus. In each case strand-specific RT primers were used to generate cDNA and PCR was performed to produce an amplicon that spans the gap in the raw annotations. Left: An IGV [53, 54] snapshot with tracks showing the gene, our raw CUT, and our final CUT annotations for the strains S288c, Σ1278b, and JAY291 after conversion to the 4-way Pecan alignment (see Methods). Additionally we show the location of each primer used and the resultant amplicon of a positive merge result. Strand-specific data is color coded with Watson/plus strand in red and Crick/minus strand in blue. Right: 2 % agarose gel showing RT-PCR results. For each candidate we designed two primer pairs with each pair located on either side of the gap between raw CUT annotations as identified by our HMM. We generated strand-specific cDNA from both WT and rrp6Δ total RNA samples with each reverse primer and performed PCR on these cDNA with F1/R2 primer pair. F1/R2 primers should produce a merge amplicon product only if the candidate CUT is a single transcript spanning the gap in raw CUT annotations. Amplification in R1 primed cDNA served as a negative control, as amplification should only occur in R1 primed cDNA; this also helped to confirm strand-specificity. We included genomic positive control, a no primer control (NPC) RT sample to distinguish false-primed cDNAs (denoted with *), and a no template control (NTC) to distinguish primer dimers. (PDF 8940 kb) [file 12864_2016_2622_MOESM1_ESM.pdf]

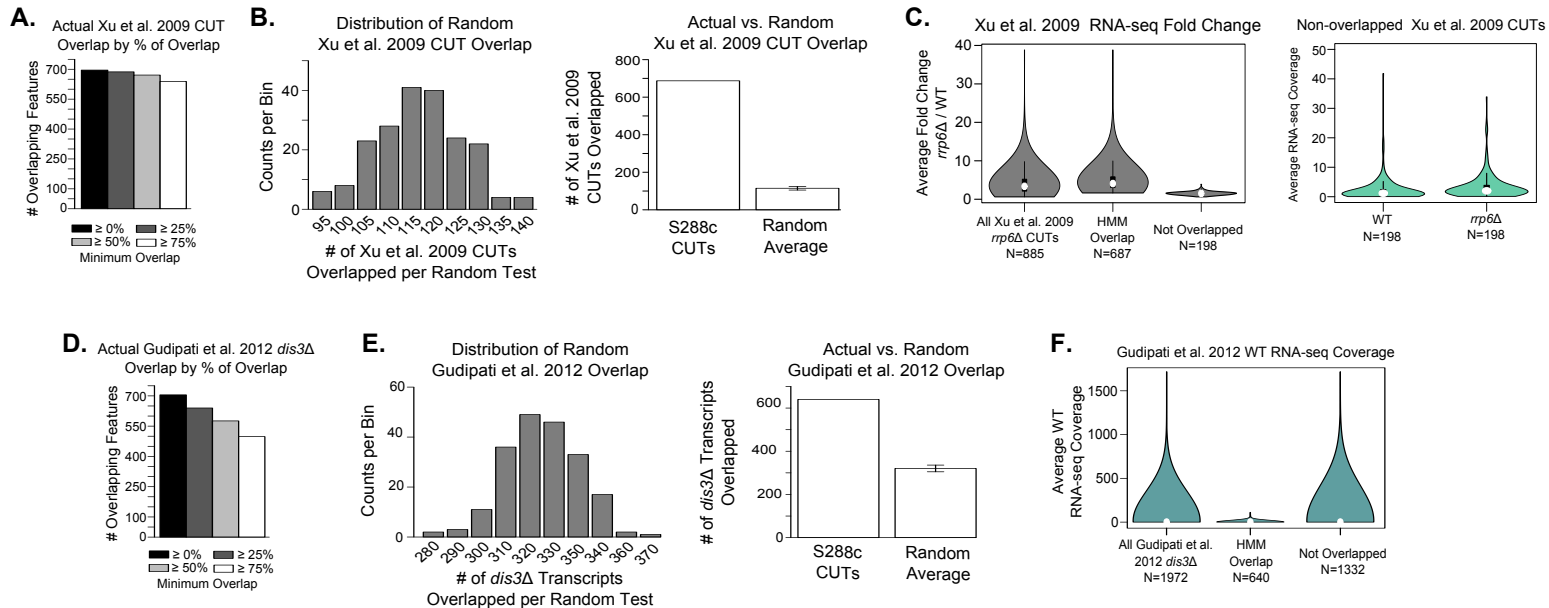

Supplement: Additional file 2: Figure S2. — S288c HMM CUT comparison to Xu et al. [11] and Gudipati et al. [31] annotations. Comparisons of S288c CUTs identified by our HMM and Xu et al. [11] CUTs or Gudipati et al. [31] dis3Δ transcripts. Extent to which minimum overlap influences number of features concordant between HMM detected CUTs and A) Xu et al. [11] CUTs. B) Overlap is more than would be expected by chance. S288c CUT annotations were randomized (see Methods) and the number of features overlapped in each data set was collected over 200 iterations and plotted as a histogram. The average number of features overlapped after 200 iterations, with error bars denoting standard deviation, is plotted for comparison to actual S288c overlap results. Actual S288c CUTs overlap is greater than 2 standard deviations from random trials. C) Violin plots as seen in Fig. 1 d showing average RNA-seq fold change for all Xu et al. [11] CUTs, Xu et al. [11] CUTs overlapped by CUT identified by our HMM, and Xu et al. [11] CUTs missed by our study where we observe equivalent expression in WT and rrp6Δ backgrounds. (D-F) Similar comparison for Gudipati et al. [31] dis3Δ transcripts. (PDF 54 kb) [file 12864_2016_2622_MOESM2_ESM.pdf]

**A.** CUT 3' Nucleosome Metagene Plot

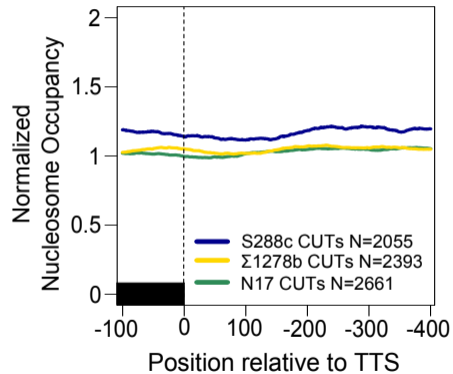

**B.** S288c 3' Nucleosome Metagene Plot

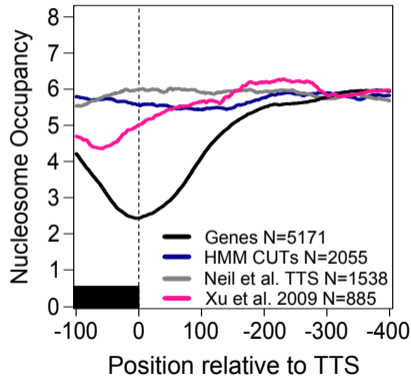

Split  
Xu et al. 2009 CUTs  
by HMM Overlap

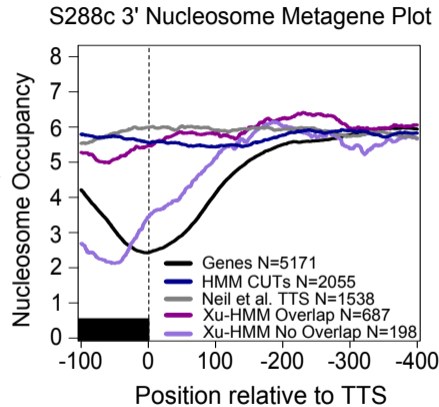

Supplement: Additional file 3: Figure S3. — CUTs appear to lack a 3′ NFR. A) Metagene plot showing the average nucleosome occupancy of a 500 bp window around the TTS of all S288c(blue), Σ1278b(yellow), and S.paradoxus N17(teal) CUTs identified by our HMM. For comparison across strains, nucleosome occupancy was normalized by the average nucleosome occupancy per base pair in each strain. Like S288c CUTs, we see do not see 3′ nucleosome depletion in our other strains for which nucleosome occupancy data is available. B) Left: Metagene plot showing the average S288c nucleosome occupancy of a 500 bp window around the TTS of all genes with a 3′ UTR annotation (black), our HMM identified CUTs (blue), Neil et al. 2009 TTS clusters (grey), and Xu et al. [11] CUTs (pink). Moderate 3′ nucleosome depletion can be seen for Xu et al. CUTs 2009. Right: When we split the Xu et al. [11] CUT annotations into two groups, those overlapped by an S288c CUT identified by our HMM (maroon), and those that are not (lilac), we see distinct nucleosome occupancy patterns for the two groups. Those Xu et al. [11] CUTs that overlap an S288c CUT identified by our HMM also appear to lack a 3′NFR and the moderate depletion previously seen in the left graph is largely restricted to those Xu et al. [11] CUTS that we failed to detect and which also appear to be stable, albeit lowly expressed RNAs (see Fig. 1d and Additional file 2: Figure S2C). (PDF 70 kb) [file 12864_2016_2622_MOESM3_ESM.pdf]

## S288c 3' Nucleosome Metagene Plot

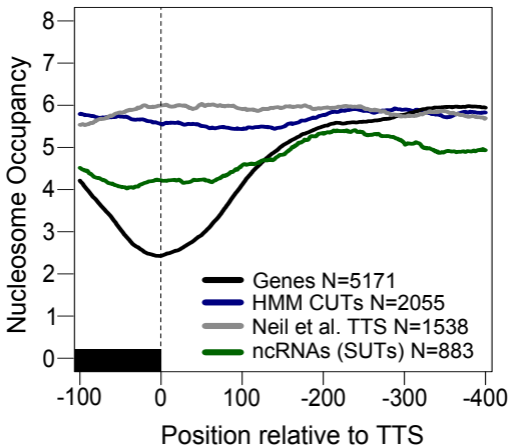

Supplement: Additional file 4: Figure S4. — ncRNAs have moderate 3′ nucleosome depletion. Metagene plot showing the average S288c nucleosome occupancy of a 500 bp window around the TTS of all genes with a 3′ UTR annotation (black), our HMM identified CUTs (blue), Neil et al. 2009 TTS clusters (grey), and ncRNAs (green) also known as stable unannotated transcripts (SUTs) from Xu et al. [11]. ncRNAs show moderate 3′ nucleosome depletion within the same 200 bp region where genes have a strong 3′ NFR producing a nucleosome occupancy pattern that is distinct from both CUTs and genes. (PDF 33 kb) [file 12864_2016_2622_MOESM4_ESM.pdf]

## 3x CUTs - qPCR Validation Results

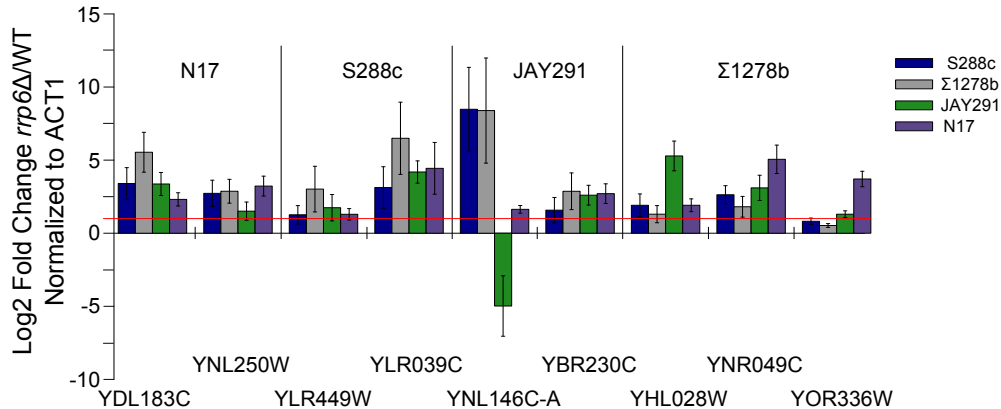

Supplement: Additional file 6: Figure S5. — Assessment of HMM false negative rate by RT-qPCR. RT-qPCR of CUTs expressed in three out of four strains (3x CUTs). For simplification candidates are named based on closest or overlapping protein-coding gene annotations (x-axis). Candidates are grouped and labeled (above the bar plot) according to the strain that lacks the corresponding CUT annotation. RT-qPCR was performed either strand-specifically or non-strand specifically depending on the presence of overlapping antisense gene annotations (see Methods; Additional file 16). Log2 fold change of rrp6Δ/WT was calculated after normalization to ACT1. The red dashed line marks two-fold cutoff. In all but one instance, JAY291 YNL146C-A, the “missing” CUT shows elevated expression, as seen in the remaining strains. All qPCR was performed with biological triplicates and error bars denote standard deviation of fold change by coefficient of variation calculations. (PDF 33 kb) [file 12864_2016_2622_MOESM6_ESM.pdf]

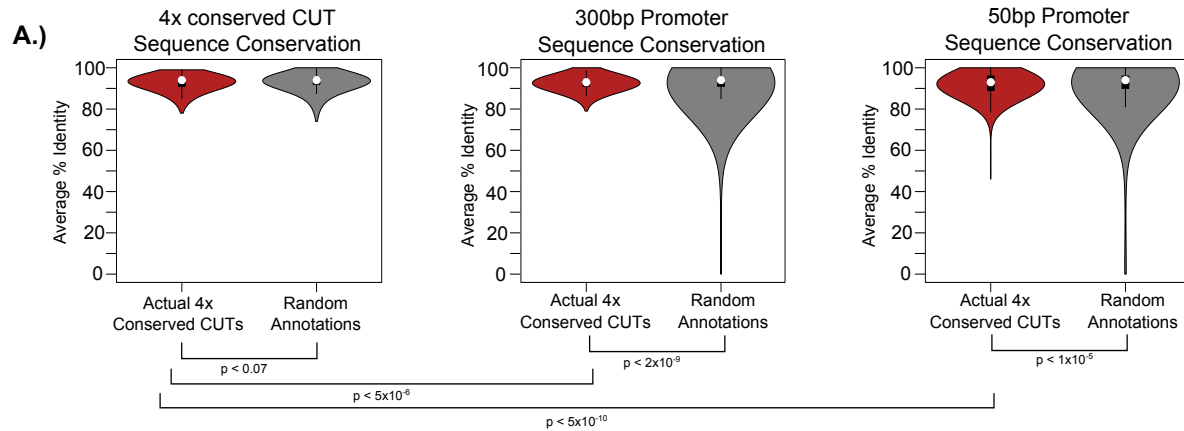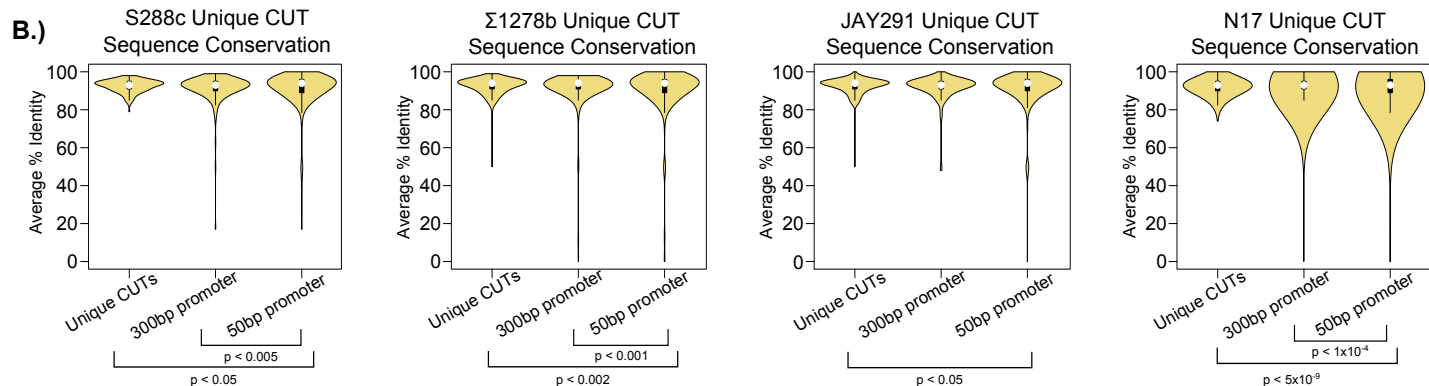

Supplement: Additional file 7: Figure S6. — Sequence conservation of CUTs. A) Violin plots showing the average sequence conservation, calculated from our 4-way genome alignment, of all 4x conserved CUTs, 300 bp upstream and 50 bp upstream promoters (red), and compared to the average percent identity of a randomized set of annotations (grey) that recapitulates the 4x conserved CUTs in length and frequency. We used the S288c start coordinate and the longest stop coordinate as the start and stop coordinates for the 4x conserved CUTs when calculating average percent identity. Included are all p-values < 0.1 obtained by the two-sided KS test. B) Violin plots showing the average sequence conservation, calculated from our 4-way genome alignment, of the CUTs unique to each strain and the 300 bp upstream and 50 bp upstream promoters. Included are all p-values < 0.1 obtained by the two-sided KS test. (PDF 56 kb) [file 12864_2016_2622_MOESM7_ESM.pdf]

# Conserved Antisense gene-CUT Pairs

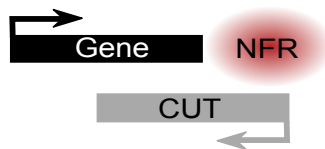

**A.**

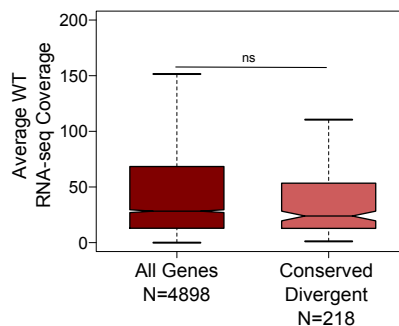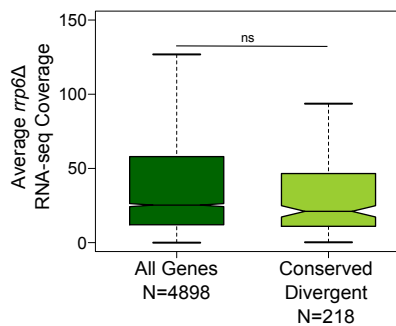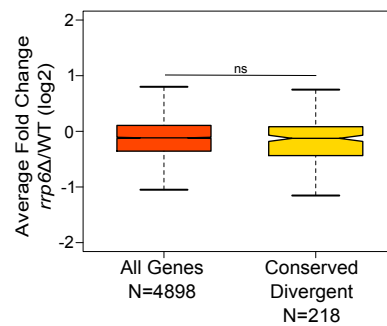

$\Sigma 1278b$

**B.**

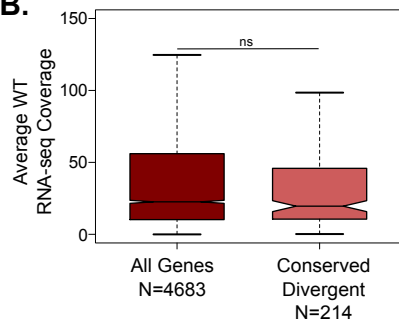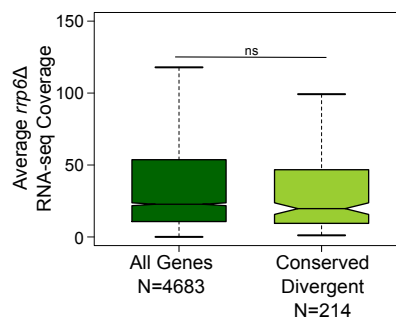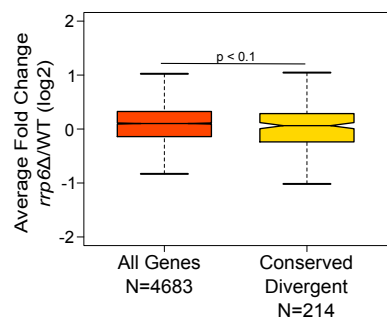

JAY291

**C.**

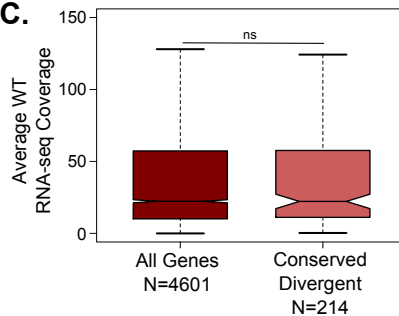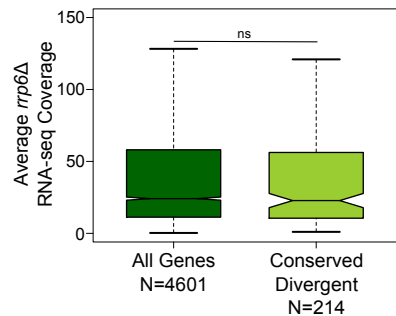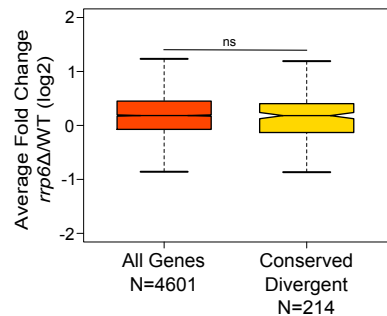

*S. paradoxus* (N17)

Supplement: Additional file 9: Figure S8. — Conserved antisense gene-CUT pairs in Σ1278b, JAY291, and S.paradoxus. Examination of antisense gene-CUT pairs containing a 4x conserved CUT. Box and whisker plots shows the distribution the average WT RNA-seq coverage (red), rrp6Δ RNA-seq coverage (green), log2 rrp6Δ/WT RNA-seq fold change (orange) for all expressed genes with a 3′ UTR annotation and the subset of genes from antisense gene-CUT pairs with a 4x conserved CUT in A) Σ1278b, B) JAY291, and C) S.paradoxus. All points outside the whiskers (outliers) are not displayed. All p-values are derived from the two-sided KS test. Nonsignificant (ns) p-value ≥ 0.1. (PDF 46 kb) [file 12864_2016_2622_MOESM9_ESM.pdf]

# Conserved Divergent gene-CUT Pairs

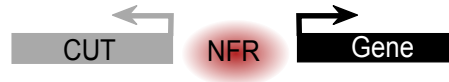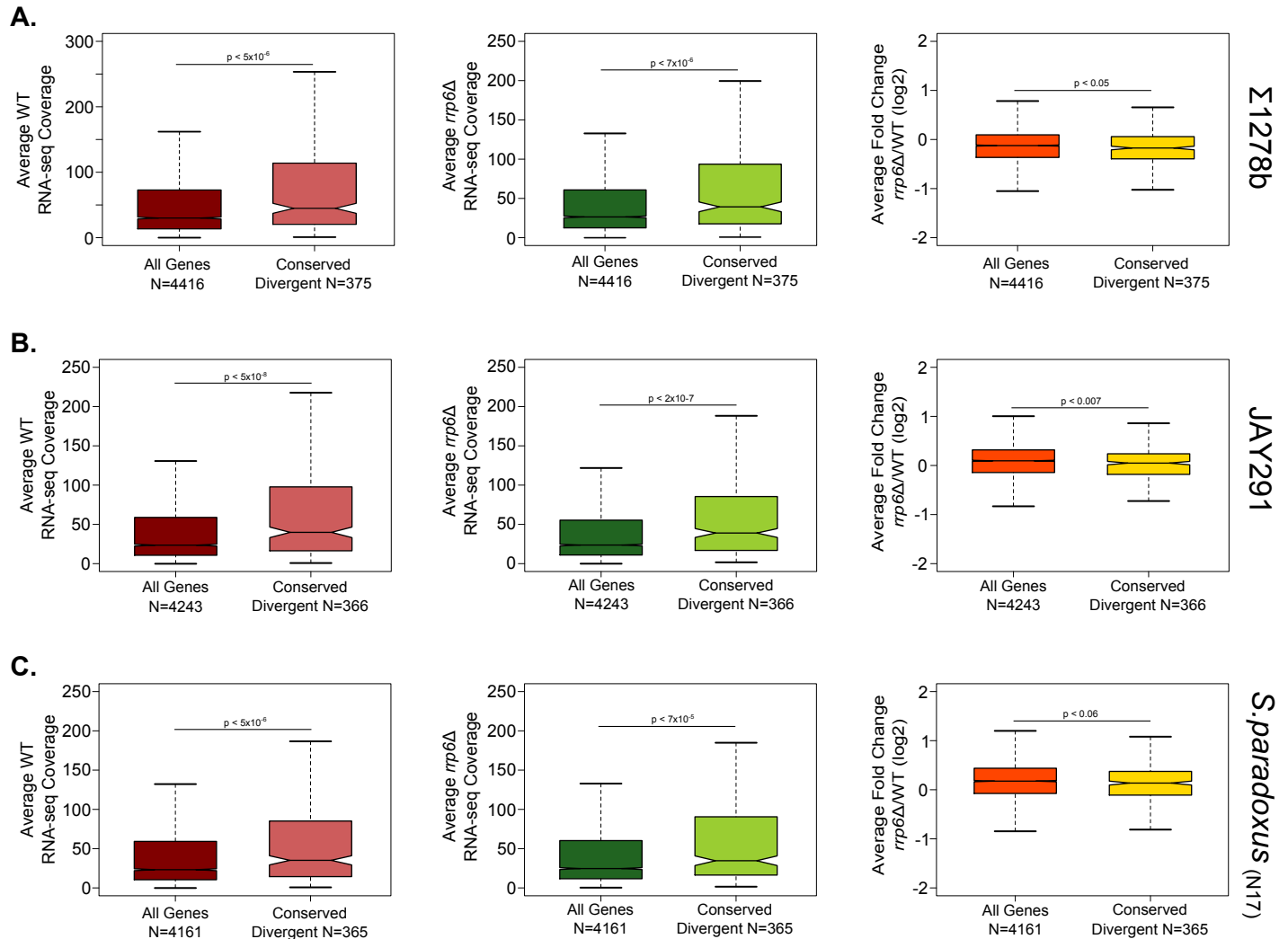

Supplement: Additional file 11: Figure S9. — Conserved divergent gene-CUT pairs in Σ1278b, JAY291, and S.paradoxus. Examination of divergent gene-CUT pairs containing a 4x conserved CUT. Box and whisker plots shows the distribution the average WT RNA-seq coverage (red), rrp6Δ RNA-seq coverage (green), log2 rrp6Δ/WT RNA-seq fold change (orange) for all expressed genes with a 5′ UTR annotation and the subset of genes from antisense gene-CUT pairs with a 4x conserved CUT in A) Σ1278b, B) JAY291, and C) S.paradoxus (N17). All points outside the whiskers (outliers) are not displayed. All p-values are derived from the two-sided KS test. (PDF 46 kb) [file 12864_2016_2622_MOESM11_ESM.pdf]

# Divergent Gene-Gene Pairs

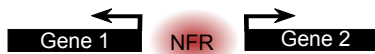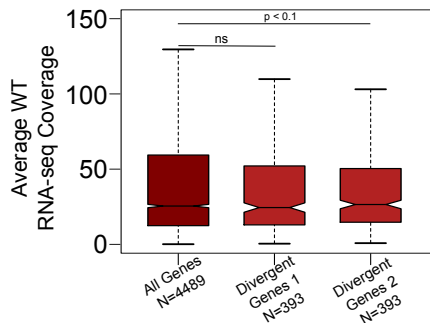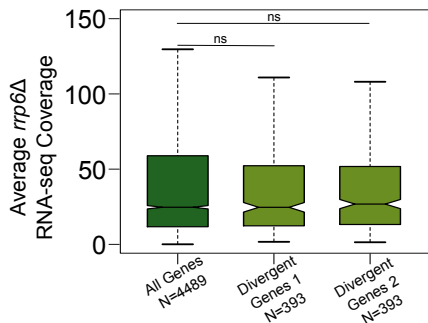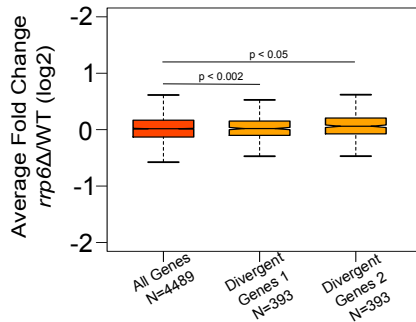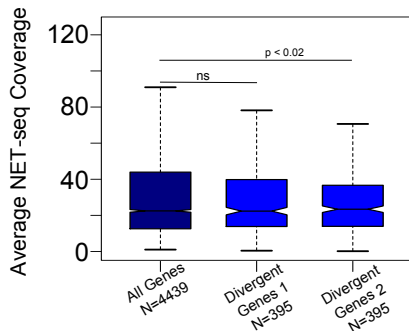

Supplement: Additional file 12: Figure S10. — Divergent gene-gene pairs in S288c. Examination of divergent gene-gene pairs in S288c. Box and whisker plots shows the distribution the average WT RNA-seq coverage (red), rrp6Δ RNA-seq coverage (green), log2 rrp6Δ/WT RNA-seq fold change (orange) for all expressed genes with a 5′ UTR annotation and the subset of genes from gene-gene pairs. Gene set 1 and gene set 2 are derived from the two separate genes from each gene-gene pair where gene 1 is also on the crick strand as shown in the schematic. All points outside the whiskers (outliers) are not displayed. All p-values are derived from the two-sided KS test. Nonsignificant (ns) p-value ≥ 0.1. (PDF 32 kb) [file 12864_2016_2622_MOESM12_ESM.pdf]

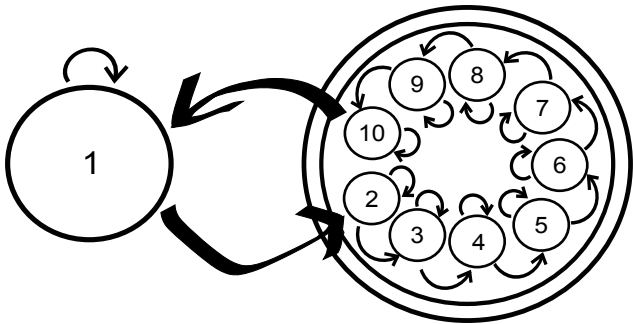

Supplement: Additional file 14: Figure S11. — 10-state explicit duration HMM. A state diagram of our explicit duration HMM showing expansion of state 2 into nine equivalent sub-states. The first state is parameterized to non-elevated regions of the transcriptome (i.e. not CUTs) and the remaining states are parameterized for elevated (approximately ≥ 2 fold) regions of the transcriptome (i.e. CUTs). We expanded the CUT state into nine identical sub-states with unidirectional movement through the model thereby setting the minimum length of a CUT to nine nucleotides and producing a 10-State model that approximates a hidden semi-Markov model [51]. (PDF 37 kb) [file 12864_2016_2622_MOESM14_ESM.pdf]

**A.** Random CUT conservation

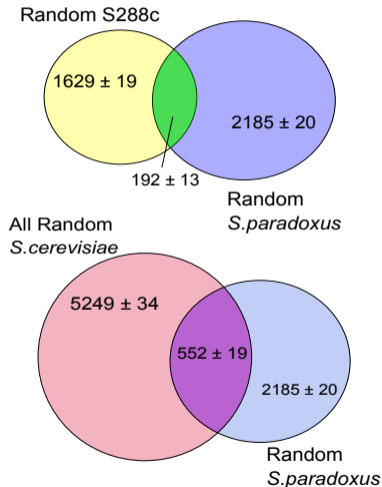

**B.** Actual vs. Random  
4x CUT conservation

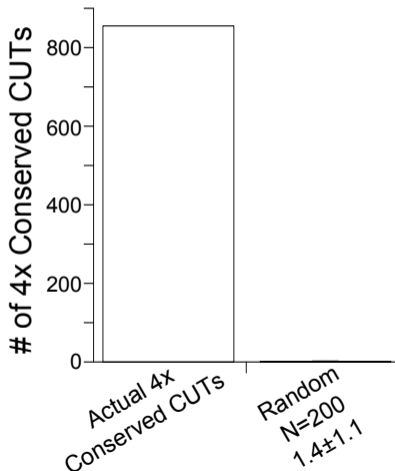

Supplement: Additional file 15: Figure S12. — Results of Randomized CUT Conservation Analysis. To determine the significance of our CUT conservation analysis we randomized CUT annotations in all four strains to assess the chance of CUT conservation simply by chance. A) Venn diagrams as seen in Fig. 3C showing the average and standard deviation of conserved CUT expression between the S.cerevisiae strain S288c and S.paradoxus (N17) and the conserved CUT expression between all S.cerevisiae strains (S288c, Σ1278b, and JAY291) and S.paradoxus (N17) after 200 randomized trials. B) Bar graph showing the actual total number of 4x conserved CUTs as found by our study and the average and standard deviation of the total number of 4x conserved CUTs after 200 randomized trials. (PDF 42 kb) [file 12864_2016_2622_MOESM15_ESM.pdf]
