# Supplementary material for: Survey of cryptic unstable transcripts in yeast
Source: BMC Genomics. 2016 Apr 26;17:305. doi: 10.1186/s12864-016-2622-5 (PMC4845318; doi:10.1186/s12864-016-2622-5)
Supplement: Additional file 8: Figure S7. — 4x conserved CUTs show increased 5′ nucleosome depletion relative to all CUTs. Metagene plot showing the average nucleosome occupancy in A)S288c, B)Σ1278b, and C)N17 of a 500 bp window around the TSS for all CUTs identified by our HMM in the respective strain (black) and all 4x conserved CUTs as annotated in each respective strain (grey). (PDF 54 kb) [file 12864_2016_2622_MOESM8_ESM.pdf]

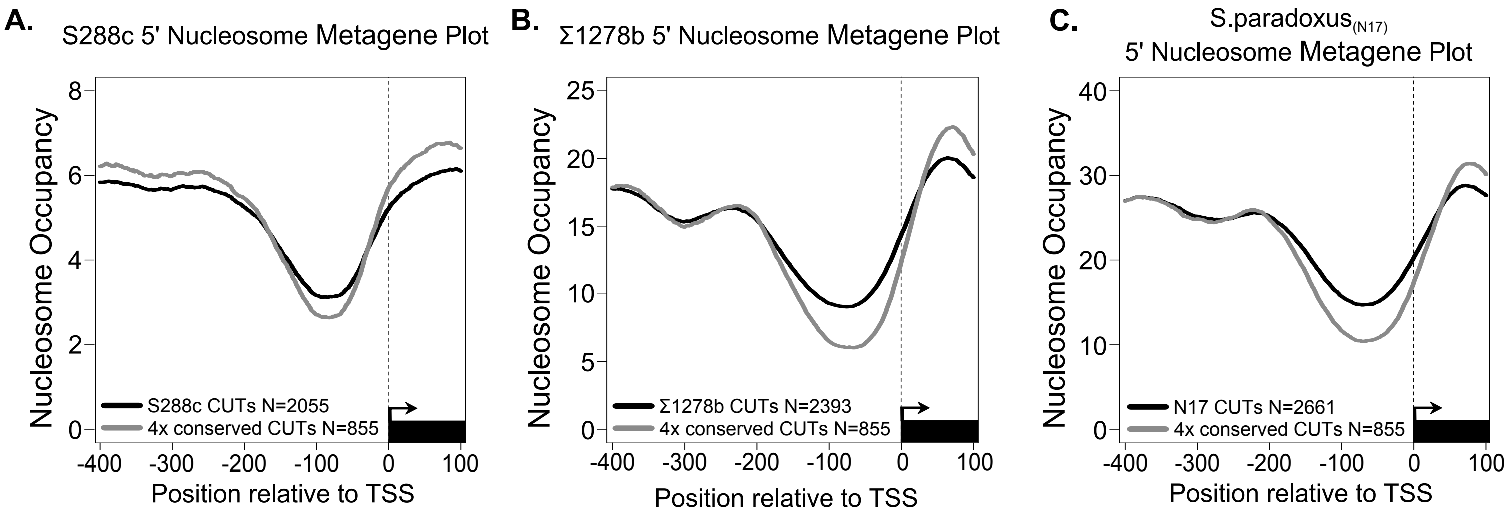
A.
S288c 5' Nucleosome Metagene Plot
B.
Σ1278b 5' Nucleosome Metagene Plot
C.
S.paradoxus(N17)
5' Nucleosome Metagene Plot
Nucleosome Occupancy
Position relative to TSS
-400
-300
-200
-100
0
100
S288c CUTs N=2055
4x conserved CUTs N=855
Σ1278b CUTs N=2393
4x conserved CUTs N=855
N17 CUTs N=2661
4x conserved CUTs N=855
